# Synchronous online lecturing or blended flipped classroom with jigsaw: an educational intervention during the Covid-19 pandemic

**DOI:** 10.1186/s12909-022-03915-5

**Published:** 2022-12-07

**Authors:** Zinat Mohebbi, Alireza Mortezaei-Haftador, Manoosh Mehrabi

**Affiliations:** 1 Department of Nursing, School of Nursing and Midwifery, Shiraz University of Medical Sciences, Shiraz, Iran; 2 School of Nursing and Midwifery, Shiraz University of Medical Sciences, Shiraz, Iran; 3 Department of e-Learning in Medical Sciences, Virtual School (Comprehensive Center of Excellence for Advanced Electronic Learning in Medical Sciences), Shiraz University of Medical Sciences, Shiraz, Iran

**Keywords:** Online systems, Communication skills, Critical thinking, Nursing students

## Abstract

**Background:**

The Covid-19 pandemic has changed the education system throughout the world. This study aimed to compare synchronous online lecturing with blended flipped classroom plus jigsaw in terms of their effects on the students’ learning, communication skills and critical thinking disposition.

**Methods:**

This is an educational intervention conducted at the Nursing and Midwifery School of Shiraz University of Medical Sciences. Two incoming students of nursing and midwifery were selected by complete enumeration. Then synchronous online lecturing was given to one group (*n* = 40) and blended flipped classroom and jigsaw to the other (*n* = 44). After that, given the prevailing conditions, both methods were performed fully online. Then Participants completed an online questionnaire. A researcher-made learning questionnaire, the Interpersonal Communication Skills Questionnaire developed by Fetro, and Ricketts’ Critical Thinking Disposition Inventory were used to assess the study variables.

**Results:**

The mean learning scores in the blended group were slightly higher but this difference was not significant (*P* = 0.767). In the blended group, the mean scores of communication skills were significantly higher after the intervention in all the dimensions, except for empathy & intimacy and listening skills. In the online lecture group, there was no significant difference between before and after the intervention. Communication skills (*P* < 0.001) scored significantly higher in the blended group after the intervention than that in the synchronous online lecturing group in all the dimensions except for empathy & intimacy. In the online lecture group, there was no significant difference in critical thinking disposition between before and after the intervention. In the blended group, the overall score of critical thinking disposition and its dimensions was significantly higher after the intervention (*P* < 0.001), except for the perfection dimension. There was no significant difference between the two groups in terms of the mean total score of critical thinking disposition and its dimensions before and after the intervention.

**Conclusion:**

Given the global circumstances, the blended method was more effective in promoting learning outcomes and communication skills than synchronous online lectures. Furthermore, it seems that this new approach could improve critical thinking.

## Background

The twenty-first century nurses are faced with many challenges in their job. They need specific skills to meet their needs today and in the future, and to improve patient care [1].

One of the skills expected of individuals in the 21st century is to establish good communications with others [2]. This skill is necessary for all individuals because it encompasses all aspects of life and career prospects [3]. Nursing is one of the professions that require the most complex set of communication skills [4]. Nursing students’ communication skills can be promoted when the educational system emphasizes the communication aspect of their education, ultimately leading to better patient care [5]. Active teaching methods such as the flipped classroom tend to set learning activities in such a way that teacher-student interactions are promoted [6].

Critical thinking is another essential attribute of individuals in today’s competitive world [7]. Critical thinking disposition describes the individual’s inclination to use critical thinking when faced with problems for the ultimate goal of performing an appropriate behaviour [7, 8]. Without critical thinking disposition, individual’s use of their intellectual abilities is incomplete [9]. To promote critical thinking, nursing schools should use methods to encourage students to look for uncertainties and develop different viewpoints than others [10]. Modern active learning methods strengthen students’ critical thinking, increase their ability to identify and assess their own learning needs, increase their decision-making power in different situations, and reinforce their problem-solving skills [11].

In general, COVID-19 has affected individual’s lives and mental health indices, including their sleep pattern and physical activity in Iran and across the world due to the anxiety it causes and the increased hours of working with computers [12–18]. Furthermore, COVID-19 has particularly had a serious effect on educational organizations, teachers and students throughout the world [19]. This disease has changed educational processes globally [20]. During the pandemic, 1.2 billion students left their classrooms to keep up with the social distancing protocols in place [21]. To continue educating their students, universities have inevitably resorted to teaching methods such as e-learning [22, 23]. Synchronous online lecturing is an e-learning method in which the professor and students engage in a synchronous online lecture [24]. Students gain the highest level of interactive experience in this method, even compared to what they would have experienced in a traditional classroom [25].

Blended learning is another educational method that can be used during a situation like the COVID-19 pandemic [26]. This method has been recommended for cases of disease outbreak, when distance is a barrier, and also for other cases in which the learners’ access to education is limited [27, 28]. The flipped classroom is one of the subclasses of this educational method in which the content is presented by videos, recorded lectures, or brief instructions to the students before attending the class in person. Afterwards, a face-to-face session is held in the classroom to review the topic through student-centred activities [29]. This method has attracted a lot of popularity in medical education in recent years [30, 31].

Collaborative learning is another active learning method that has a marked standing in education [3]. The jigsaw technique is one of the subclasses of collaborative learning in which a specific part of the topic is assigned to each learner to study and learn. With this technique, the learners are first obliged to profoundly study and understand the topic and then share it with the other learners working on the same topic. Next, learners from each specific group, who are responsible for a distinct part of the topic, come together and present their part of the topic to members of the other groups [32]. Using technology alongside teaching by jigsaw can enhance teaching and learning [33].

The review of literature showed that nearly all the studies in this field, such as the review study by Tang et al. (2018), have found that medical students are highly satisfied with online lectures and show good progress afterwards [34]. In another study, Kim et al. (2018) argued that flipped classroom is an effective teaching and learning method to enhance the knowledge and performance of nursing students [35]. Buhr et al. (2014) also reported jigsaw as an effective learning method for medical students [36].

Furthermore, Dewi (2020) showed that online learning positively affects the learners’ communication skills [37]. Sudin et al. (2021) argued that jigsaw is an effective method for improving learners’ communication skills [38]. A study conducted by Tathahira (2020) concluded that the online learning environment is effective in improving learners’ critical thinking [39]. In another study, it was reported that jigsaw learning positively affects critical thinking [40]. Moreover, in another study, the flipped classroom method had a positive effect on 21st -century skills, such as communication and critical thinking [41]. Nevertheless, researchers have recommended further studies on the effect of flipped classroom on critical thinking and problem-solving skills [42].

Educating undergraduate nursing students must be fundamentally transformed with the use of modern teaching techniques to improve the quality of education and train graduates ready for today’s complex nursing practices [43]. Given the above, the present study compared two influential learning methods during the COVID-19 crisis to determine which approach is better. The two methods include synchronous online lectures and blended flipped classroom with collaborative jigsaw. This study compared these two methods in terms of their effect on the students’ learning, interpersonal communication skills, and critical thinking disposition, which are considered essential 21st -century skills. As a new educational effort in line with the pandemic conditions, the blended flipped classroom and jigsaw method was performed fully online.

## Methods

### Study design

The present educational intervention study was conducted in 2020 on two groups of undergraduate nursing students. Synchronous online lecturing was compared to blended flipped classroom and jigsaw in terms of their effects on learning basic nursing concepts, communication skills and critical thinking disposition among undergraduate nursing admissions of September 2019 and January 2020.

By complete enumeration, the undergraduate nursing students admitted to the School of Nursing and Midwifery of Shiraz University of Medical Sciences in September 2019 and January 2020 were selected as the study subjects. The inclusion criteria were: 1. Being in the second semester of the undergraduate nursing program of Shiraz School of Nursing and Midwifery; 2. Willingness to take part in the study; and 3. Taking the course on basic nursing concepts. The study exclusion criteria were: 1. Returning incomplete questionnaires; and 2. More than one session absence. Of the 48 September admissions, 42 entered the synchronous online lecture group, and of the 55 January admissions, 47 entered the blended group, all by giving their informed consent. The study began after obtaining the university research deputy’s permission and approval for the project as well as approval from the Ethics Committee of Shiraz University of Medical Sciences (IR.SUMS.REC.1399.199).

### Intervention

The present study was conducted fully online with some help from the Virtual School of Shiraz University of Medical Sciences (Centre of Excellence for e-Learning in Medical Sciences). To assign the students to the synchronous online lecture group and the blended flipped classroom and jigsaw group, the names of both the September and January admissions were written on two pieces of paper and tossed in a bag, and the first name drawn out of the bag was assigned to the synchronous online lecture method. A person not involved in the study then pulled out one of the pieces of paper out of the bag, which turned out to be the list of the September admissions, and these students were thus assigned to the synchronous online lecture group, and the January 2020 admissions were automatically assigned to the blended method. At the beginning of the study, the September admissions were briefed on the methodology of the research, and informed consent forms and questionnaires (including the demographic questionnaire, the interpersonal communication skills questionnaire, and the critical thinking disposition inventory) were electronically distributed among the students through Porsline website. Nine sessions of training in basic nursing concepts were provided to the students through synchronous online lectures based on the lesson plan. The synchronous online lectures were held in Adobe Connect. Similar to conventional lectures, the PowerPoint presentation designed for each session was presented during the sessions and the instructor (the first author) gave a lecture and interacted with the students. After each session, the PowerPoint presentation for that session was given to all the students to keep. Out of the 42 students in this group, two were excluded due to being absent too many sessions. After the ninth session of the synchronous online lectures, the questionnaires were re-completed by the students. As in the synchronous online lecture group, the blended flipped classroom and jigsaw group (i.e., the January admissions) was also briefed on the research methods and was given informed consent forms plus questionnaires to complete. Given the pandemic situation, these sessions were held fully online. A week before each session, a video clip covering the relevant topic plus a number of questions prepared by the same instructor from the synchronous online lecture group were prepared in Camtasia software and uploaded onto the Learning Management System. Before each session, the students had one week to watch the recorded video clips or prepare themselves through other sources. In addition to fully studying the relevant session’s topic, each student was assigned a specific topic from the whole subject to study further in depth. On the day of the online class, Breakout Rooms were formed in Adobe Connect and the students with common specific topics entered their own dedicated group. All the groups had 20 min to share and exchange information about their specific topics with their other group members, and the same instructor from the synchronous online lecture group joined the groups and monitored their work and answered the students’ questions. Afterwards, the students joined their original groups, and each student represented their common group, i.e., the group in which they had learnt about a specific common topic, and shared information on that topic and gave a summary of the specific topic to the other members of the class. Finally, the instructor also summarized the entire subject and answered any questions. Out of the 47 students in the blended learning group, three were excluded due to being absent too many sessions. After the ninth session, the questionnaires were completed again by the students. The final exam scores were used to assess the students’ learning in both groups.

### Study variables and questionnaires

First, the students’ demographic details, including age, gender, academic year, and GPA were recorded.

In this research, learning was defined as the mean scores obtained by the participants of both the synchronous online lecture and blended learning groups in the nine sessions of training on basic nursing concepts. A researcher-made questionnaire was used to assess the students’ learning. The questionnaire contained the final exam questions for the basic nursing concepts course, including 32 multiple-choice questions. Face and content validity were assessed by five experienced faculty members who taught the subject, and the tool’s validity was confirmed. Reliability was confirmed using a test-retest carried out on ten students from each group, with Spearman’s correlation coefficient showing a very high correlation between the test and retest scores (synchronous online lecture group: *R* = 0.934, *P* < 0.001, blended method group: *R* = 0.946, *P* < 0.001).

Interpersonal communication skills were determined by the score obtained in the Interpersonal Communication Skills Questionnaire developed by Fetro. This 65-item questionnaire is scored based on a five-point Likert scale. The validity and reliability of this questionnaire had formerly been confirmed by Fetro and Rhodes [44]. Mahmoodi et al. translated and implemented this questionnaire for the first time in Iran in 2016. The exploratory factor analysis performed on the 65 items of the questionnaire resulted in fewer items and more components. The number of items was reduced to 54 and the number of components was increased to six, with the addition of assertiveness and listening skills. The validity and reliability of this questionnaire were confirmed in the same article, and this version can be used for assessing interpersonal communication skills [45].

Critical thinking was defined as the score obtained on the Critical Thinking Disposition Questionnaire designed in 2003 by Ricketts in the US. This tool is a self-reporting questionnaire that measures critical thinking disposition with 33 statements in three subscales, as follows: Innovation, perfection and commitment. The face and content validity and reliability of this questionnaire had formerly been confirmed by Ricketts [46, 47]. In Iran, the validity and reliability of this questionnaire were confirmed by Pakmehr [48].

### Statistical analysis

After the students completed the questionnaires, data was analysed in IBM SPSS Statistics for Windows (Version 25.0. Armonk, NY: IBM Corp). The parametric quantitative and categorical variables are presented as mean ± SD and frequency (percentage), respectively. The normal distribution of the quantitative data was checked by the Shapiro-Wilk test for normality. *P*-values greater than 0.05 were considered normally distributed. Differences between the groups at baseline and the end of the study were evaluated using an independent sample t-test for the parametric variables (if the assumption of normality and equal variances were met). Analysis of covariance (ANCOVA) was also applied (after checking the assumptions, including the independence of observations, homogeneity of variances and normality of dependent variable) to compare changes in the variables between the two groups during the study after controlling for potential confounding factors. A Chi-square test was conducted to compare the categorical variables between the two groups. Besides, a paired-samples t-test was used to assess the within-group changes for all the quantitative variables. Spearman’s correlation coefficient was used to analyse the correlation between the test and retest scores. The significance level was set at *P* < 0.05.

**Fig. 1:**
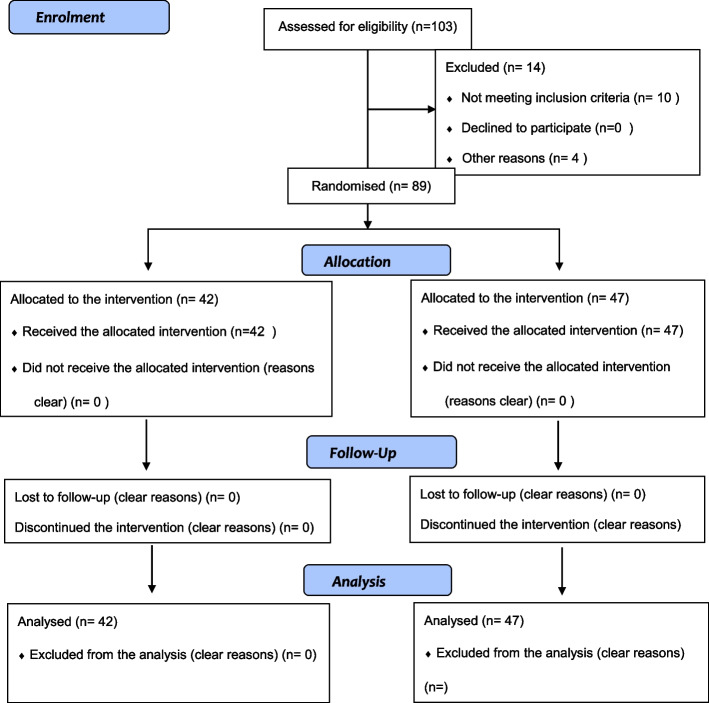
The students’ recruitment flow diagram

Figure 1 shows the recruitment of the participants in the two groups.

## Results

### Demographic

Out of the 84 participating students, 40 learners were in the synchronous online lecture group and 44 in the blended group. The majority of the participants were female in both groups (synchronous online lecture: 21 women; blended group: 23 women). The mean age was 20.88 ± 3.164 years and the mean GPA 14.574 ± 1.947 in the synchronous online group and 21.30 ± 3.122 years and 17.005 ± 0.770 in the blended group. No significant differences were found between the two groups in terms of gender (*P* = 0.983) and age (*P* = 0.542), but there was a significant difference between them in terms of their GPA (*P* < 0.001) (Table 1).

**Table 1:** Demographic characteristics of the nursing students in the two groups

Variable	Online lecture	Blended method	*P*-Value
Age, Mean ± SD	20.88 ± 3.164	21.30 ± 3.122	0.542^a^
Mean GPA, Mean ± SD	14.574 ± 1.947	17.005 ± 0.770	< 0.001^a^
*Gender*			
* Male, Frequency (percentage)*	21 (52.5)	23 (52.3)	0.983^b^
Female, Frequency (percentage)	19 (47.5)	21 (47.7)	

^a^ Based on the independent-samples t-test

^b^ Based on the Chi-square test

### Learning assessment

The assessment of the mean learning scores in the two groups showed that the mean score was only slightly higher in the blended group (19.499 ± 0.766) than that in the synchronous online lecture group (16.629 ± 0.692), with significant differences between them (*P* < 0.001).

### Interpersonal communication skills

In the synchronous online lecture group, no significant difference was found in the total communication skills scores and the scores of its dimensions before and after the intervention. In the blended group, however, the mean scores of communication skills and its dimensions were higher after the intervention, and the difference was significant in all the dimensions except for empathy and intimacy (*P* = 0.150) and listening skills (*P* = 0.392) (Table 2).

Before the intervention, no significant difference was found between the two groups in the mean scores of communication skills and its dimensions, with the exception of assertiveness (*P* = 0.009). After the intervention, the mean scores of communication skills and its dimensions were higher in the blended group compared to the synchronous online lecture group, with a significant difference between them in all the dimensions except for empathy and intimacy (*P* = 0.140) (Table 2). Given the significant pre-intervention differences between the two groups in terms of assertiveness, to investigate the effect of the intervention on this variable and control the pre-intervention scores, the ANCOVA test was used for the comparison of the two groups. The results showed that the changes were higher in the blended group (6.318 ± 7.379) than those in the synchronous online lecture group (-0.850 ± 3.076), with a significant difference between them (*P* < 0.001).

**Table 2:** The within- and between-group comparison of the scores in the Interpersonal Communication Skills (ICS) Questionnaire before and after the intervention

ICS Dimensions	Online lecture			Blended method			Between group	
	**Before**	**After**	***P*** **-Value**^**w**^	**Before**	**After**	***P*** **-Value**^**w**^	***P*** **-Value**^**b**^	***P*** **-Value**^**a**^
Empathy & Intimacy	57.675 ± 5.757	57.525 ± 6.679	0.857	58.727 ± 5.558	59.954 ± 5.057	0.150	0.710	0.140
Assertiveness	30.800 ± 4.045	29.950 ± 3.761	0.088	26.886 ± 4.199	33.204 ± 7.410	< 0.001	0.009	0.008
Communication Skills	31.500 ± 3.967	32.200 ± 4.403	0.220	31.863 ± 4.289	34.863 ± 3.770	< 0.001	0.633	0.005
Conflict Resolution	24.450 ± 4.326	24.600 ± 4.683	0.803	25.613 ± 4.081	27.750 ± 5.176	0.025	0.094	0.009
Maintain & Develop	28.825 ± 3.492	28.950 ± 3.565	0.777	28.045 ± 5.304	30.954 ± 3.766	< 0.001	0.936	0.015
Listening Skills	15.025 ± 2.877	14.375 ± 2.923	0.057	15.977 ± 2.435	16.340 ± 1.916	0.392	0.426	0.049
Total	188.275 ± 16.084	187.600 ± 17.021	0.593	187.113 ± 15.344	203.068 ± 17.829	< 0.001	0.862	< 0.001

*P*-Value^w^: The within-group comparison in each group

*P*-Value^b^: The between-group comparison before the intervention

*P*-Value^a^: The between-group comparison after the intervention based on the ANCOVA after controlling the effect of GPA

### Critical thinking disposition

In the synchronous online lecture group, the results showed no significant difference in critical thinking disposition and its dimensions before and after the intervention. Nonetheless, in the blended group, the mean overall score of critical thinking disposition and its dimensions was higher after the intervention compared to before, as the difference was significant in all the dimensions except for perfection (*P* = 0.108) (Table 3).

The results showed no significant differences between the two groups in terms of the mean scores of critical thinking disposition and its dimensions before the intervention. Nonetheless, the statistical analysis of the data after the intervention showed that the mean total score of critical thinking disposition and its dimensions was higher in the blended method group than that in the synchronous online lecture group, although not in a statistically significant way (Table 3).

**Table 3:** Comparison of the two groups regarding the mean score of Critical Thinking Disposition (CTD) and its dimensions

CTD Dimensions	Online lecture			Blended method			Between group	
	**Before**	**After**	***P*-Value**^w^	**Before**	**After**	***P*** **-Value**^w^	***P*** **-Value** ^b^	***P*** **-Value** ^a^
Creativity, Mean ± SD	42.700 ± 4.339	43.600 ± 4.929	0.168	43.500 ± 4.702	45.931 ± 3.762	0.002	0.759	0.179
Perfection, Mean ± SD	29.750 ± 3.578	30.550 ± 3.644	0.229	30.590 ± 3.661	31.909 ± 3.502	0.108	0.811	0.251
Commitment, Mean ± SD	49.200 ± 5.160	49.900 ± 6.428	0.384	50.000 ± 5.400	52.840 ± 5.455	0.002	0.995	0.113
Total, Mean ± SD	121.650 ± 9.149	124.050 ± 12.268	0.083	124.090 ± 10.458	130.681 ± 9.070	< 0.001	0.953	0.071

*P*-value^w^: The within-group comparison in each group

*P*-value^b^: The between-group comparison before the intervention

*P*-value^a^: The between-group comparison after the intervention based on the ANCOVA after controlling the effect of GPA

## Discussion

The present study was conducted to investigate two new educational methods used extensively during the COVID-19 pandemic with respect to their effects on the students’ learning, communication skills and critical thinking disposition.

The demographic results showed no significant differences between the two groups in terms of gender and age, but there was a significant difference between them in terms of mean GPA, which was higher in the blended group than that in the synchronous online lecture group.

In this study, attempts were made to ensure that the educational technique used would be the only factor affecting the results. Selecting students admitted in the same semester and dividing them into two groups could have led to communication bias between the groups and affected the results in both groups; therefore, two completely different admissions had to be chosen, which caused this difference in GPAs. Another important reason was that the September admission students (the synchronous online lecture method) had taken all their previous semester’s exams in person, which made cheating very unlikely, while the January admission group (the blended method) had taken all their previous semester’s exams online, which made cheating and obtaining a higher GPA much more likely.

Regarding the students’ learning, the results showed that the mean final exam scores in the blended flipped classroom and jigsaw group were slightly higher than those in the synchronous online lecture group; however, both methods led to high scores in basic nursing principles and concepts, indicating the effectiveness of both methods on the students’ learning. In one study, Yuliza (2019) argued that jigsaw learning is an effective method for improving students’ learning [49]. In another study, Tang et al. (2020) stated that blending the flipped classroom method with online classes can be more efficient than online classes alone [50]. In the present study, both methods were highly effective in terms of the students’ learning. As a result, when the blended method cannot be applied, synchronous online lectures can be used to improve students’ learning.

According to the present findings, the blended method affected the students’ interpersonal communication, while synchronous online lectures had no such effects. In their study, Popa et al. (2020) stated that communication is poor in online learning [51]. In another study conducted during the COVID-19 pandemic, Istiqomah et al. (2021) argued that online jigsaw leads to improvements in students’ communication skills [52]. In a study conducted before this pandemic, Williams et al. (2018) argued that blended flipped classroom and jigsaw can increase collaboration with the learners [53]. The class atmosphere created with the blended method provided the students with the opportunity to have greater interactions and collaboration with one another as well as with their professors. Moreover, the students interacted and collaborated more when placed in small groups, which was not the case in synchronous online lectures.

According to the within-group comparisons, the given intervention had a significant effect on critical thinking in the blended learning group, while this effect was not significant in the synchronous online lecture group. Despite the higher mean scores of critical thinking disposition and all its dimensions in the blended group compared to the synchronous online lecture group, the intergroup difference was not significant. In another study, Zuriah and Rahmandani (2021) showed that online education during the COVID-19 pandemic promotes students’ critical thinking [54]. In a study conducted before the pandemic, Williams et al. (2018) stated that blended flipped classroom and jigsaw enhance critical thinking [53]. In another study, Yen (2020) showed that online education using the flipped classroom method during COVID-19 encourages students to reflect more on the class material before joining the class, and group discussions further help with it, too [55]. In the blended method described, the students have access to the class material plus some questions before joining the class, which further enables them to think about the class material. Moreover, group discussions and the instructor’s sufficient time for creating challenging scenarios for the students with regard to the subject matter encourage the students to reflect more deeply on the class subject.

### Limitations

Given the COVID-19 pandemic and the resultant circumstances and the online nature of the stages of the study, the researchers did not test the students’ learning before beginning the intervention. Consequently, the students may have stored the questions in their devices, which could have led to false results; therefore, further studies are recommended on this subject. Furthermore, our participants were entered into the university in different semesters. Therefore, studies with larger sample size of students in same-semester are recommended.

## Conclusion

Since the Covid-19 pandemic conditions may have permanently changed the course of education, it is imperative to adopt the best method to meet all the students’ needs. In the present study, efforts were made to design a blended approach of flipped classroom plus jigsaw that offered more student-centred educational activities and promoted skills that the students would need for their future careers. The results showed the superiority of the blended method over a simple online teaching method in terms of learning outcomes and communication skills. Furthermore, the blended method had possible roles in improving critical thinking. Accordingly, this method can be used during the COVID-19 pandemic and for other distance learning purposes as an appropriate educational method.

## Data Availability

The data that support the findings of this study are available from the corresponding author, [M.M], on a special request.
